# Comparing the contents of patient-reported outcome measures for fatigue: EORTC CAT Core, EORTC QLQ-C30, EORTC QLQ-FA12, FACIT, PRO-CTCAE, PROMIS, Brief Fatigue Inventory, Multidimensional Fatigue Inventory, and Piper Fatigue Scale

**DOI:** 10.1186/s12955-024-02316-0

**Published:** 2024-12-02

**Authors:** Maria Rothmund, Micha J. Pilz, Nathalie Egeter, Emma Lidington, Claire Piccinin, Juan I. Arraras, Mogens Groenvold, Bernhard Holzner, Marieke van Leeuwen, Morten Aa. Petersen, John Ramage, Heike Schmidt, Teresa Young, Johannes M. Giesinger, on behalf of the EORTC Quality of Life Group

**Affiliations:** 1 Department of Psychiatry, Psychotherapy, Psychosomatics, and Medical Psychology, BA, University Clinic of Psychiatry II, Innsbruck Medical University, Anichstraße 35, Innsbruck, A-6020 Austria; 2 Institute of Psychology, University of Innsbruck, Innrain 52, Innsbruck, A-6020 Austria; 3 Cancer Prevention Trials Unit, Queen Mary University of London, Empire House, 67-75 New Rd, London, E1 1HH UK; 4 Quality of Life Department, EORTC, Avenue E. Mounier, 83/11, Brussels, 1200 Belgium; 5 Medical Oncology Department, Hospital Universitario de Navarra, C/Irunlarrea 3, Pamplona, ES-31008 Spain; 6 Palliative Care Research Unit, Department of Geriatrics and Palliative Medicine GP, Bispebjerg & Frederiksberg Hospital, Bispebjerg bakke 23, Copenhagen, DK-2400 Denmark; 7 University of Copenhagen, Copenhagen, DK-1353 Denmark; 8 Department of Psychiatry, Psychotherapy, Psychosomatics, and Medical Psychology, Innsbruck Medical University, University Clinic of Psychiatry I, Anichstraße 35, Innsbruck, A-6020 Austria; 9 Division of Psychosocial Research & Epidemiology, The Netherlands Cancer Institute, Plesmanlaan 121, 1066 CX, Amsterdam, SO22 4NR The Netherlands; 10 Department Gastroenterology, Hampshire Hospitals Foundation Trust, University of Winchester, Sparkford Rd, Winchester SO22 4NR, Hampshire, RG24 9NA UK; 11 University Clinic and Outpatient Clinic for Radiotherapy and Institute of Health and Nursing Science, Medical Faculty, Martin Luther University Halle-Wittenberg, Halle (Saale), DE-06108 Germany; 12 Lynda Jackson Macmillan Centre, Mount Vernon Cancer Centre, Northwood, Middlesex, Rickmansworth Rd, HA6 2RN UK

**Keywords:** Cancer, Oncology, Fatigue, Patient-reported outcome measures, Content analysis, QLQ-C30, FACIT, PROMIS, EORTC CAT, PRO-CTCAE

## Abstract

**Background:**

To assess fatigue in cancer patients, several patient-reported outcome measures (PROMs) are available that differ in content. To support the selection of suitable measures for specific applications and to evaluate possibilities of quantitative linking, the present study provides a content comparison of common fatigue measures, scales, and item banks. We included the EORTC CAT Core, EORTC QLQ-FA12, EORTC QLQ-C30, FACIT-F, PROMIS Fatigue (Cancer item bank v1.0), Brief Fatigue Inventory (BFI), Multidimensional Fatigue Inventory (MFI-20), Piper Fatigue Scale (PFS-12), and PRO-CTCAE.

**Methods:**

All items of the included measures were linked to the International Classification of Functioning, Disability and Health (ICF). Additionally, they were categorized as assessing general, physical, emotional, or cognitive fatigue. Descriptive statistics were used to display the contents covered in each measure and to allow for a qualitative comparison.

**Results:**

The measures consist of 160 items in total and covered primarily contents of the ICF components ‘Body functions’, ‘Activities and participation’, and ‘Environmental Factors’. Most ICF codings refer to ‘b1300 Energy level’ (9–67% of the codings per instrument; 47% of all coded content). Within the broad categorization of types of fatigue, most items were classified as general fatigue (33–100% of the codings per instrument; 49% of the overall item pool). While the EORTC CAT Core focuses exclusively on physical and general fatigue, FACIT and BFI additionally assess emotional fatigue. The EORTC QLQ-FA12, PROMIS, MFI-20, and PFS-12 cover all fatigue components, including cognitive fatigue.

**Discussion:**

The review provides an in-depth content comparison of PROMs assessing cancer-related fatigue. This can inform the selection of suitable measures in different clinical contexts. Furthermore, it will inform quantitative analyses to facilitate comparison of scores obtained with different PROMs.

**Supplementary Information:**

The online version contains supplementary material available at 10.1186/s12955-024-02316-0.

## Background

Fatigue is one of the most common symptoms of cancer and its treatment, that affects about half of the cancer patients [1] and persists for years after treatment completion in many cancer survivors [2]. Cancer-related fatigue is defined as ‘a distressing, persistent, subjective sense of physical, emotional, and/or cognitive tiredness or exhaustion related to cancer or cancer treatment that is not proportional to recent activity and interferes with usual functioning’ [3]. It is more severe, persistent and debilitating compared to normal tiredness and not relieved by sleep or rest [4]. Thus, it has a profound effect on patients’ health-related quality of life, limiting their ability to participate in work, social life and activities of daily living [5]. Furthermore, fatigue commonly occurs together with depression and can be difficult to disentangle due to overlapping symptoms, such as loss of energy, sleep disturbance, and loss of interest [6].

Previous studies have shown that clinicians consistently underestimate fatigue levels among cancer patients [7, 8], which highlights the need for robust patient-reported outcome measures (PROMs) for this key symptom that, in fact, is only accessible from the patients’ own experience. Currently, many validated PROMs are available and some of them are widely used in cancer patients. These measures, that are in part cancer-specific, differ in content, i.e., regarding aspects of fatigue they measure, and questionnaire length. While some measures focus only on fatigue, others are multidimensional questionnaires that also include a fatigue scale.

This variety of available PROMs allows for flexibility in choosing the optimal measure for a specific setting or purpose, but also makes comparisons of results from different clinical studies challenging and limits the possibilities of conducting meta-analyses or interpreting data combined in systematic reviews. To overcome these challenges, there are different methods to convert scores on the metric of one measure to the metric of another [9, 10] by establishing so-called ‘cross-walks’ based on methods such as regression analysis [11], equipercentile equating [12], or item response theory modelling [13]. However, alongside the use of statistical methods to convert PROM scores, attention should also be paid to the content assessed, as crosswalks between measures assessing different concepts may have limited meaningfulness [11]. Thus, content analyses of PROMs are needed to inform about which combinations of PROMs are conceptually similar and may be linked through quantitative methods.

As part of a larger project by the European Organization for Research and Treatment of Cancer (EORTC) Quality of Life Group, we are currently exploring the potential to convert and compare scores from commonly used PROMs in cancer research to the computer adaptive testing (CAT) item banks by the EORTC, i.e., the EORTC CAT Core [14, 15]. This work includes a qualitative content comparison followed by quantitative analyses on the actual linking of scores. The qualitative results of these content analyses of PROMs have been published previously for the physical functioning [16], emotional functioning [17], and role/social functioning domains [18].

In this article, we describe the qualitative content comparison of PROMs assessing fatigue in cancer patients. We followed a standard method to categorize the contents of all items into the International Classification of Functioning, Disability, and Health [19, 20] and additionally classified all items as referring to physical, cognitive, emotional, or general fatigue.

## Methods

### Comparator measures

Our content analysis included PROMs measuring fatigue that are frequently used in cancer clinical trials or recommended for fatigue assessment in cancer patients [21, 22]. Short descriptions of these PROMs are provided in Table 1. The following nine PROMs were included in their validated English-language versions:

**Table 1:** Description of PROMs included in the content analysis

**Name**	**Scale/s(number of items)**	**Recall period**	**Response format**	**Target population**	**Score range and scoring**
EORTC CAT Core Fatigue item bank	Single scale (34)	Last week	Four-point scale ranging from ‘not at all’ to ‘very much’	Cancer	T-scores
EORTC QLQ-FA12	Physical (5) Emotional (3) Cognitive (2) Interference (1) Support (1)	Last week	Four-point scale ranging from ‘not at all’ to ‘very much’	Cancer	0 – 100 (sum scores with linear transformation)
EORTC QLQ-C30 Fatigue scale	Single scale (3)	Last week	Four-point scale ranging from ‘not at all’ to ‘very much’	Cancer	0 – 100 (sum scores with linear transformation)
PROMIS Cancer Fatigue item bank v1.0	Single scale (54)	Last week	Five-point scale ranging from ‘Never’ to ‘Always’, ‘Not at all’ to ‘Very much’, or ‘None’ to ‘Very’	Cancer	T-scores
FACIT Fatigue Scale v4	Single scale (13)	Last week	Five-point Likert scale ranging from ‘Not at all’ to ‘Very much’	Cancer	0 – 52 (sum score)
Brief Fatigue Inventory (BFI)	Single scale (9)	various versions	11-point scale ranging from ‘No fatigue’ to ‘As bad as you can imagine’ or ‘Does not interfere’ to ‘Completely interferes’	Generic	0 – 10 (mean score)
Multidimensional Fatigue Inventory (MFI-20)	General (4) Physical (4) Reduced motivation (4) Reduced activity (4) Mental fatigue (4)	“Lately”	Five-point scale ranging from ‘yes, that is true’ to ‘no, that is not true’	Generic	4 – 20 (sum score)
NCI PRO-CTCAE fatigue items	Severity (1) Interference (1)	Last week	Five-point Likert scales ranging from ‘None’ to ‘Very severe’ or ‘Not at all’ to ‘Very much’	Cancer	0 – 4 (raw item scores, separately)
Piper Fatigue Scale (PFS-12)	Behavioural/severity (3) Affective meaning (3) Sensory (3) Cognitive/mood (3)	Past four weeks	11-point Likert scale ranging from ‘no fatigue’ to ‘worst fatigue possible’	Cancer	0 – 10 (mean score)

- EORTC CAT Core Fatigue item bank [23]
- EORTC QLQ-FA12 [24]
- EORTC QLQ-C30 Fatigue scale [25] 
- PROMIS Fatigue Cancer item bank v1.0 [26]
- FACIT Fatigue Scale (v4) [27]
- Brief Fatigue Inventory (BFI) [28]
- Multidimensional Fatigue Inventory (MFI-20) [29]
- NCI PRO-CTCAE items for fatigue [30]
- Piper Fatigue Scale (PFS-12) [31].

## Content analysis using the International Classification of Functioning, Disability, and Health (ICF)

The World Health Organization International Classification of Functioning, Disability, and Health (ICF) is a hierarchical framework for health-related aspects covering body functions (b), activities and participation (d), and body structures (s) but also environmental (e) and personal factors (p). The first, second, third and fourth level domains are sub-categories of these components, labelled with additional digits. Fatigue-related examples are provided in Table 2. In this way, the ICF offers ‘codes’ to categorise various health aspects. PROMs usually cover body functions (b) or activities and participation (d) and sometimes refer to environmental contexts (e), whereas body structures (s) and personal factors (p) are less relevant.

**Table 2:** Examples for the hierarchical structure of the ICF [24]

**Domain**	**Code**	**Title**	**Description**
**Component**	**b**	**Body functions**	
First level (chapter)	b4	Functions of the cardiovascular, haematological and respiratory systems	This chapter is about the functions involved in the cardiovascular system (functions of the heart and blood vessels), the haematological and immunological systems (functions of blood production and immunity), and the respiratory system (functions of respiration and exercise tolerance).
Second level	b455	Exercise tolerance functions	Functions related to respiratory and cardiovascular capacity as required for enduring physical exertion.
Third level	b4552	Fatiguability	Functions related to susceptibility to fatigue, at any level of exertion.
**Component**	**b**	**Body functions**	
First level (chapter)	b1	Mental functions	This chapter is about the functions of the brain: both global mental functions, such as consciousness, energy and drive, and specific mental functions, such as memory, language and calculation mental functions.
Second level	b130	Energy and drive functions	General mental functions of physiological and psychological mechanisms that cause the individual to move towards satisfy specific needs and general goals in a persistent manner.
Third level	b1300	Energy level	Mental functions that produce vigour and stamina.
**Component**	**d**	**Activities and Participation**	
First level (chapter)	d2	General tasks and demands	This chapter is about general aspects of carrying out single or multiple tasks, organizing routines and handling stress. These items can be used in conjunction with more specific tasks or actions to identify the underlying features of the execution of tasks under different circumstances.
Second level	d230	Carrying out daily routine	Carrying out simple or complex and coordinated actions in order to plan, manage and complete the requirements of day-to-day procedures or duties, such as budgeting time and making plans for separate activities throughout the day.
Third level	d2301	Managing daily routine	Carrying out simple or complex and coordinated actions in order to plan and manage the requirements of day-to-day procedures or duties.

A standard methodology to apply the codes to PROMs has been developed and refined by Cieza et al. [19, 20]. The steps include first identifying all meaningful concepts per item and second assigning the concepts’ corresponding ICF codes. An item can be assigned more than one code if it contains several meaningful concepts (e.g., ‘Have you *become tired* from *walking upstairs*?’). If no suitable code exists, items are coded as ‘not covered’ (nc) or ‘not definable’ (nd). These can be further specified as relating to the overall health conditions (nc-hc) or quality of life (nc-qol).

The ICF provides two third level categories for fatigue, i.e. b1300 Energy level and b4552 Fatiguability (see Table 2). For this analysis, we used category b1300 when items covered the level of tiredness or energy (e.g., ‘How fatigued were you on average?’), whereas b4552 was used for items describing the susceptibility to getting tired (e.g., ‘Have you become tired from walking upstairs?’).

All items were coded by two independent reviewers from a pool of five authors with previous experience in ICF coding (NE, MR, EL, CP, JG). A third reviewer from the same pool of reviewers was consulted if agreement could not be reached. Interrater agreement is presented as total agreement (%) for second level codes [32].

### Classification of items as measuring general, physical, cognitive, or emotional fatigue

To align with recent conceptualisations of cancer-related fatigue as a multi-dimensional construct comprised of physical, emotional, and cognitive components [33], we further categorized all items into these components. Each item was classified based on its content or the contextual information. For example, an item asking about fatigue when exercising would be classed as physical (e.g., ‘Have you become tired from walking up stairs?’), while an item asking about fatigue while reading would be classed as cognitive (e.g., ‘How often were you too tired to watch television?’). The component emotional fatigue contains items on fatigue interfering with motivation or affects (e.g., feeling ‘too tired to feel happy’ or ‘listless’). An item would be classed as general if it included fatigue that could have multiple components (e.g., ‘I am able to do my usual activities’ or ‘Have you become tired from carrying out your duties and responsibilities’) or if no descriptive or contextual information was given (e.g., ‘How fatigued were you on average?’ or ‘Have you lacked energy?’). All items related to sleep were coded as physical fatigue as this was considered to be a physiological aspect. Unspecific items asking about being ‘tired’, ‘lack of energy’, or ‘feeling worn-out’ were classified as ‘general’; except ‘weak’ was classified as physical, which is consistent with previous methodological approaches [34]. Items were classified as ‘other’ if they could not be coded (e.g., ‘Have other people noticed your fatigue?’).

Again, all items were coded by two independent reviewers from the pool of five authors (NE, MR, EL, CP, JG). Each item could only be assigned one classification. A third reviewer was consulted if agreement could not be reached. Interrater agreement is presented as total agreement (%).

### Data analysis

The ICF codes and types of fatigue covered in each included measure are presented descriptively with absolute and relative frequencies to support content comparisons. For the ICF coding, one item could have more than one code since an item could consist of more than one meaningful concept. For the additional categorization, each item was only assigned to one of the components, i.e., general, physical, cognitive, or emotional fatigue.

## Results

### Content comparison based on the ICF framework

For all nine of the investigated PROMs, the majority of ICF codings were within the component “b - Body functions”, followed by the component “d - Activities and participation”. Table 3 outlines the number of ICF codings per level for all included measures. The component “b - Body functions” covered 100% of the content of the EORTC QLQ-C30 fatigue scale, followed by the PFS-12 (80%), the EORTC QLQ-FA12 (79%), the PROMIS Fatigue Item Bank (71%), the BFI (71%), the PRO-CTCAE (67%), EORTC CAT Core Fatigue (65%), the FACIT-Fatigue (60%) and the MFI-20 (50%). The component “d - Activities and participation” was most frequently coded for the FACIT-Fatigue (35%), the PRO-CTCAE (33%), the EORTC CAT Core Fatigue (31%), the PROMIS Fatigue Item Bank (29%), the BFI (29%), the PFS-12 (20%), the EORTC QLQ-FA12 (14%), and the MFI-20 (14%). In “e - Environmental factors” the EORTC QLQ-FA12 had 7% of its codings and the FACIT-Fatigue 5%. Content that was not definable in the ICF represented 4% of the codings of the EORTC CAT Core Fatigue and 36% of the MFI-20.

**Table 3:** Overview of the ICF codings of the PROMs under investigation

	**EORTC CAT Core Fatigue**	**EORTC QLQ-FA12**	**EORTC QLQ-C30 Fatigue**	**PROMIS Item Bank – Fatigue**	**FACIT Fatigue**	**BFI**	**MFI-20**	**NCI PRO-CTCAE**	**PFS-12**
Items in scale	34	12	3	54	13	10	20	2	12
Items covered by ICF	34	12	3	54	13	10	12	2	12
Unique first-level categories	8	3	1	10	6	5	4	2	3
Unique second-level categories	15	5	1	17	8	7	7	2	7
Unique third-level categories^a^	17	5	1	22	10	8	8	2	7
Number of third-level codings assigned^a^	50	14	3	91	20	17	14	3	15
Number of codings as not covered (nc) or not defined (nd)	2	0	0	0	0	0	8	0	0

^a^If no third level category was available, the number of second level categories was counted. Codings as not covered (nc) or not definable (nd) were not included in this count

Regarding third-level categories, content of the EORTC CAT Core was coded most frequently in “b1300 Energy level (42%), “b4552 Fatiguability” (17%), and “d2301 Managing daily routine” (8%). All three items of the EORTC QLQ-C30 fatigue scale were exclusively linked to the third level category ‘b1300 Energy Level’. For the EORTC QLQ-FA12 the most frequent third-level categories were in “b1300 Energy level (43%), “b1528 Emotional functions, other specified” (17%), “d2301 Managing daily routine” and “b1608 Thought functions, other specified” (both 14%).

For the PROMIS Fatigue Item Bank the category “b1300 Energy level” covered 56% of the content, with all other third-level categories representing at most 4% of the content.

The FACIT-Fatigue consisted of content mostly coded in “b1300 Energy level” (35%), “b1528 Emotional functions, other specified” (15%), and “d2301 Managing daily routine” (15%).

For the BFI “b1300 Energy level” covered 59% of the codings, while all other used third-level categories had 1 coding (6%).

The MFI-20 included content related to “b1400 Sustaining attention” (18%), and “b1300 Energy level”, “b1301 Motivation”, and “b4552 Fatiguability” (all 9%).

Content of the PRO-CTCAE could be coded in only two third-level categories: “b1300 Energy level” (67%) and “d2301 Managing daily routine” (33%).

**Fig. 1:**
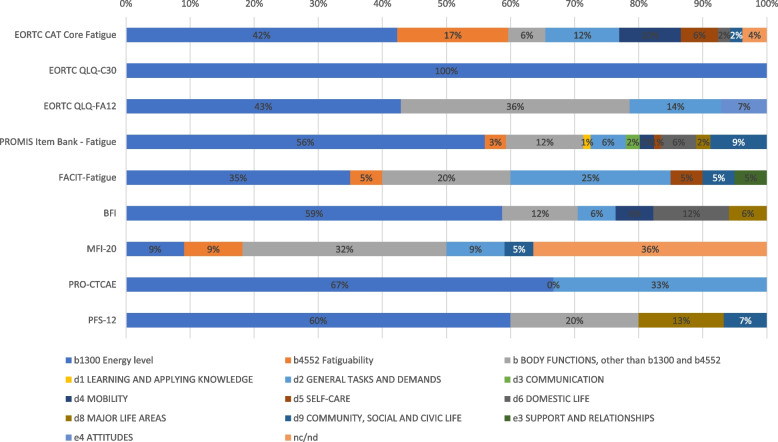
Proportion of different codings from the International Classification of Functioning, Disability, and Health (ICF) assigned to each instument

The PFS-12 consisted of content in “b1300 Energy level” (60%) and single codings (i.e., 7%) in six further categories. An overview of ICF codings assigned to each instrument is provided in Fig. 1, further details can be found in Supplementary Table S1.

Further, for the EORTC QLQ-FA12, the MFI-20, the PRO-CTCAE, and the PFS-12 the ICF categories were linked to each scale of these instruments. Details regarding the ICF coverage of each scale can be found in Supplementary Material (Table S2 and Figure S1).

The agreement between the raters at the second level was 135 out of 160 classifications (Inter-rater agreement of 84.4%).

### Content comparison for general, physical, emotional, and cognitive fatigue

Across all PROMs, half of all items were categorized as assessing general fatigue (78/160 items, 48.8%), while another 28.1% (45/160) were assigned to physical fatigue. Cognitive (21/160, 13.1%) and emotional fatigue (15/160, 9.8%) were assessed less frequently. One item (1/160, 0.6%) was categorized as ‘other’, since it does not ask about fatigue itself but about feeling understood by others (EORTC QLQ-FA12, item 12, ‘Did you feel that your tiredness is (was) not understood by the people who are close to you?’).

The 34 items of the EORTC CAT Core Fatigue item bank were assigned to physical (21/34, 61.8%) and general (13/34, 38.2%) fatigue, which is reflected in the EORTC QLQ-C30 fatigue scale as well (66.6% physical fatigue; and 33.3% general fatigue). The EORTC QLQ-FA12 assesses emotional (3/12, 25.0%) and cognitive (2/12, 16.7%) fatigue besides general (4/12, 33.3%) and physical (2/12, 16.7%) fatigue. Beyond that, the one item on feeling understood which was categorized as ‘other’ (1/12, 8.3%) takes a social issue related to fatigue into account.

The PROMIS item bank v1.0 for fatigue mostly covers general fatigue (29/54, 53.7%) but also has a focus on cognitive fatigue (13/54, 24.1%), which takes the second largest proportion followed by physical (9/54, 16.7%) and emotional (3/54, 5.6%) fatigue.

The MFI-20 has seven items on general fatigue (7/20, 35.0%), followed by five items on physical (5/20, 25.0%) and four on cognitive and emotional fatigue, respectively (each 4/20, 20.0%). The PFS-12 mainly covers general fatigue (7/12, 58.3%) as well, but also covers all other three components, i.e., physical, cognitive (each 2/12, 16.7%), and emotional (1/12, 8.3%).

The FACIT Fatigue Scale (v 4) and the BFI have no items on cognitive fatigue, but assess general (FACIT: 8/13, 61.5%; BFI: 7/10, 70.0%), physical (FACIT: 3/13, 23.1%; BFI: 1/10, 10.0%) and emotional (FACIT: 2/13, 15.4%; BFI: 2/10, 20.0%) aspects of fatigue.

**Fig. 2:**
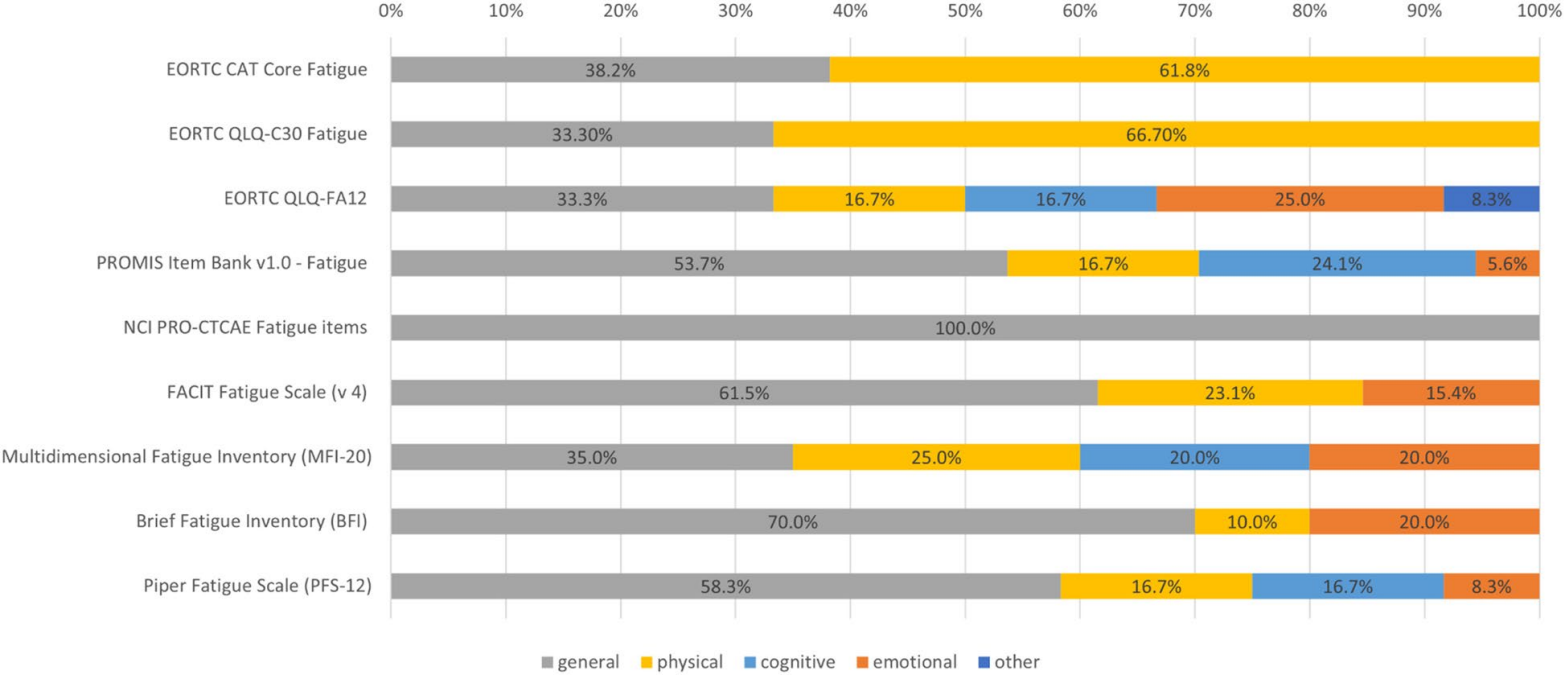
Items covering general, physical, cognitive, and emotional fatigue per instrument

Figure 2 displays the proportion of items per questionnaire that cover general, physical, emotional, and cognitive fatigue.

Further, for the EORTC QLQ-FA12, the MFI-20, the PRO-CTCAE, and the PFS-12 the types of fatigue were classified for each scale of these instruments. Details regarding the scale-specific fatigue classification can be found in the Supplementary Material (Table S3 and Figure S2).

During classification, the reviewers initially agreed on 112 of 160 items, resulting an overall agreement of 70.0%. Most conflicts (42/48, 87.5%) occurred because one reviewer classified the item as general while the other chose one of the specific fatigue components.

## Discussion

This analysis presents a comprehensive overview and comparison of fatigue-related contents assessed in several PROMs that are commonly used in cancer patients. Within the ICF framework, the most commonly used coding was ‘b1300 Energy level’. It accounted for one to two thirds of codings for almost all measures (35–67%), except the MFI-20 (9%). Beyond that, up to a third of codings per measure referred to the component ‘d – Activities and participation’ (14–35%). This indicates that the measures commonly assess the interference of fatigue with patients’ social life, role functioning, and daily living. The component “b4552 Fatiguability” was covered by a total of 15 items (6.3% of codings) across all measures, whereby the EORTC CAT comprises 9 of these 15 items. The most common d-code across all measures was ‘d2301 Managing daily routine’ with 24% (15/63 d-codings). This category refers to “the requirements of day-to-day procedures or duties” [35] and the corresponding items used generic wordings like ‘usual activities’, ‘daily activities’, or ‘completing things’. Other d-codings refer more clearly to role functioning in specific areas of life like remunerative work (d850, d859), education (d839), work at home (d6), or recreation and leisure (d920). Nevertheless, activities in all these areas can include physical (e.g., carrying bags, or sports), cognitive (e.g., planning or analytical thinking), and emotional functions (e.g., emotional work).

In our additional categorization, most items assessing interference with daily life were therefore classified as general. Thus, general fatigue takes the largest proportion in almost all questionnaires, ranging from 33.3% in the EORTC QLQ-FA12 up to 100% of fatigue-related PRO-CTCAE items. This indicates that the questionnaires differ in how many and which fatigue components they measure. It is to expect that the possibilities to establish crosswalks are best for measures covering the same fatigue components. The EORTC CAT Core Fatigue item bank mainly covers physical fatigue aspects (61.8%), only complemented by general fatigue items (38.2%). This focus is in line with the goal of the EORTC CAT Core item banks to assess the same contents as the EORTC QLQ-C30 [23]. The EORTC-QLQ-FA12 additionally contains emotional and cognitive fatigue to equal parts and one item about perceived social support (feeling understood). The PROMIS Fatigue item bank (v1.0), MFI-20, and PFS-12 also cover all four types of fatigue, whereas the FACIT Fatigue scale and BFI do not assess cognitive fatigue aspects.

In general, our analysis did not take the scale structures of multidimensional fatigue measures into account since they are not only based on content-related considerations but also on statistical methods such as factor analysis. This led to some discrepancies between our categorization and the intended scale structure of instruments. For example, the EORTC QLQ-FA12 was developed to cover only physical, cognitive, and emotional fatigue but not general fatigue [23, 24]. The item ‘I feel slowed down’ belongs to the cognitive domain within this tool. In our review, however, we classified it as general since it could refer to being cognitively or physically slowed down. Such discrepancies reflect conceptual overlaps that also become apparent during PROM development. For example, the EORTC QLQ-FA12 items on difficulties ‘getting things started’ or ‘completing things’ were initially designed to cover cognitive fatigue but in the factor analysis they were more closely related to the physical fatigue domain [24].

A large number of PROMIS items including those assessing fatigue have previously been linked to the ICF by Tucker et al. [36] and our results are mostly consistent with their codings. Most differences occurred due to our approach differentiating between ‘energy level’ as the state of being fatigued and ‘fatiguability’ as the susceptibility to fatigue.

### Limitations

The comparison of contents is always depending on the classification systems applied. With the ICF linking, we relied on a well-established approach. Nevertheless, it has been criticised before for being limited in some areas, especially emotional health [17]. As discussed above, it also provides only two categories specific to fatigue, i.e., energy level and fatiguability. These two categories provide an important distinction between the state of being fatigued and the susceptibility to fatigue. In the hierarchical ICF framework, these categories are, however, part of different first-level categories that contextualise “energy level” as a mental state and “fatiguability” as a physical state, which would not allow to classify content related to physical energy level or mental fatiguability. In our analysis, we, therefore, interpreted these two third-level categories more broadly and independent from their respective first-level categories, and used the categories fatiguability and energy level for both, physical and mental states. With an additional categorization of items assessing general, physical, cognitive, or emotional fatigue, we provide a second classification of the contents assessed by the different measures. While this provides a perspective with the conceptualization of fatigue as suggested by the NCCN [33], we could not rely on a previously established methodology for linking the content to these categories.

All reviewers involved in coding and categorizing items have a scientific background in PRO research in cancer patients and experience with ICF codings as suggested by Cieza et al. [19, 20]. To avoid confirmation bias, standardised methods were applied [19, 20], two independent ratings were collected and the item list did not display information on the subscales for the multidimensional measures.

Our study is a descriptive comparison and provides in-depth information about the contents covered by the included measures. It does not provide an evaluation of which contents are most appropriate for a specific patient population or setting and did not aim at evaluating the content validity of the PROMs. A profound evaluation of the content validity would require further information on patients’ and experts’ perception of whether the different contents are relevant, comprehensive, and comprehensible [37].

Finally, only a selection of PROMs could be included in the present content analysis. To cover the most relevant measures, we selected the measures based on the frequency of their use and on recommendations made in systematic review [21, 22].

### Clinical implications

In the clinical context, it can be challenging to select the most suitable measure. The decision requires a clear idea of the outcomes of interest as well as a profound understanding of the content covered by different measures. For example, a more comprehensive and elaborate measure should be chosen in studies explicitly focusing on fatigue outcomes, while a shorter general scale can be used if fatigue is only a secondary or exploratory outcome. Furthermore, it can be difficult to differentiate emotional and cognitive fatigue from depressive symptoms [6, 38].

Besides these direct clinical implications, our analysis will inform quantitative analyses to establish crosswalks, which can facilitate meta-analyses of studies that used different questionnaires. Thus, it will help to grade the evidence for clinical interventions more comprehensively. On the long run, this methodological approach could therefore help to improve clinical treatment and care.

## Conclusion

Previous research has shown that patient- and clinician-reports on cancer-related fatigue differ substantially [7, 8], possibly reflecting that fatigue is an experience best accessible from the patient’s perspective. The importance of fatigue in the field of cancer research is reflected by the considerable number of PROMs, which provides the advantage of being able to select the most suitable measure for different contexts, populations, and purposes, but also complicates the comparability of results. Our content comparison can inform both – selecting the right measure and comparing results. Results have shown similarities and differences between commonly used fatigue measures. While all the instruments assess the component “Energy level” and some also cover “fatiguability”, there is a broad variation of ICF categories and components assessed. The MFI-20 appears to be conceptually most distinct from the other fatigue measures, with least codings for ‘b1300 Energy level’ and most contents coded as ‘not covered’ (nc) or ‘not definable’ (nd) within the ICF. This may result from a stronger reference of the item content to generic concepts (e.g. fitness, performance level) than found in the other measures. Selecting the most suitable measures depends on the focus of the research question, while crosswalks are expected to work best between measures covering similar fatigue components. Ongoing quantitative research will further investigate the possibility to establish crosswalks between measures investigated in this article.

## Supplementary Information

Supplementary Material 1.

## Data Availability

No datasets were generated or analysed during the current study.
