# Supplementary material for: Comparing the contents of patient-reported outcome measures for fatigue: EORTC CAT Core, EORTC QLQ-C30, EORTC QLQ-FA12, FACIT, PRO-CTCAE, PROMIS, Brief Fatigue Inventory, Multidimensional Fatigue Inventory, and Piper Fatigue Scale
Source: Health Qual Life Outcomes. 2024 Dec 2;22:104. doi: 10.1186/s12955-024-02316-0 (PMC11613840; doi:10.1186/s12955-024-02316-0)
Supplement: Supplementary file 1 — Supplementary Material 1. [file 12955_2024_2316_MOESM1_ESM.docx]

**Supplementary Material**

**Table S1: Absolute and relative frequency of ICF codes linked to the PROMs under investigation.**

| **Code** | **Label** | **EORTC CAT Core Fatigue** | | **EORTC  QLQ-FA12** | | **EORTC QLQ-C30 Fatigue** | | **PROMIS Item Bank - Fatigue** | | **FACIT-Fatigue** | | **BFI** | | **MFI-20** | | **NCI PRO-CTCAE** | | **PFS-12** | |
| --- | --- | --- | --- | --- | --- | --- | --- | --- | --- | --- | --- | --- | --- | --- | --- | --- | --- | --- | --- |
|  |  | **N** | **%** | **N** | **%** | **N** | **%** | **N** | **%** | **N** | **%** | **N** | **%** | **N** | **%** | **N** | **%** | **N** | **%** |
|  | **TOTAL ICF codings** | **52** | **100%** | **14** | **100%** | **3** | **100%** | **91** | **100%** | **20** | **100%** | **17** | **100%** | **22** | **100%** | **3** | **100%** | **15** | **100%** |
| b | BODY FUNCTIONS | 34 | 65% | 11 | 79% | 3 | 100% | 65 | 71% | 12 | 60% | 12 | 71% | 11 | 50% | 2 | 67% | 12 | 80% |
| b1 | MENTAL FUNCTIONS | 23 | 44% | 11 | 79% | 3 | 100% | 62 | 68% | 11 | 55% | 12 | 71% | 9 | 41% | 2 | 67% | 12 | 80% |
| b130 | b130 Energy and drive functions | 22 | 42% | 6 | 43% | 3 | 100% | 51 | 56% | 7 | 35% | 10 | 59% | 4 | 18% | 2 | 67% | 9 | 60% |
| b1300 | b1300 Energy level | 22 | 42% | 6 | 43% | 3 | 100% | 51 | 56% | 7 | 35% | 10 | 59% | 2 | 9% | 2 | 67% | 9 | 60% |
| b1301 | b1301 Motivation |  |  |  |  |  |  |  |  |  |  |  |  | 2 | 9% |  |  |  |  |
| b134 | b134 Sleep functions | 1 | 2% |  |  |  |  |  |  | 1 | 5% |  |  |  |  |  |  |  |  |
| b1348 | b1348 Sleep functions, other specified | 1 | 2% |  |  |  |  |  |  | 1 | 5% |  |  |  |  |  |  |  |  |
| b140 | b140 Attention functions |  |  |  |  |  |  |  |  |  |  |  |  | 4 | 18% |  |  |  |  |
| b1400 | b1400 Sustaining attention |  |  |  |  |  |  |  |  |  |  |  |  | 4 | 18% |  |  |  |  |
| b144 | b144 Memory functions |  |  |  |  |  |  | 3 | 3% |  |  |  |  |  |  |  |  | 1 | 7% |
| b1449 | b1449 Memory functions, unspecified |  |  |  |  |  |  | 3 | 3% |  |  |  |  |  |  |  |  | 1 | 7% |
| b152 | b152 Emotional functions |  |  | 3 | 21% |  |  | 3 | 3% | 3 | 15% | 2 | 12% | 1 | 5% |  |  | 1 | 7% |
| b1528 | b1528 Emotional functions, other specified |  |  | 3 | 21% |  |  | 3 | 3% | 3 | 15% | 1 | 6% | 1 | 5% |  |  | 1 | 7% |
| b1529 | b1529 Emotional functions, unspecified |  |  |  |  |  |  |  |  |  |  | 1 | 6% |  |  |  |  |  |  |
| b160 | b160 Thought functions |  |  | 2 | 14% |  |  | 5 | 5% |  |  |  |  |  |  |  |  | 1 | 7% |
| b1600 | b1600 Pace of thought |  |  |  |  |  |  | 2 | 2% |  |  |  |  |  |  |  |  |  |  |
| b1608 | b1608 Thought functions, other specified |  |  | 2 | 14% |  |  | 3 | 3% |  |  |  |  |  |  |  |  |  |  |
| b1609 | b1609 Thought functions, unspecified |  |  |  |  |  |  |  |  |  |  |  |  |  |  |  |  | 1 | 7% |
| b4 | FUNCTIONS OF THE CARDIOVASCULAR, HAEMATOLOGICAL, IMMUNOLOGICAL AND RESPIRATORY SYSTEMS | 9 | 17% |  |  |  |  | 3 | 3% | 1 | 5% |  |  | 2 | 9% |  |  |  |  |
| b455 | b455 Exercise tolerance functions | 9 | 17% |  |  |  |  | 3 | 3% | 1 | 5% |  |  | 2 | 9% |  |  |  |  |
| b4552 | b4552 Fatiguability | 9 | 17% |  |  |  |  | 3 | 3% | 1 | 5% |  |  | 2 | 9% |  |  |  |  |
| b7 | b7 NEUROMUSCULOSKELETAL AND MOVEMENT-RELATED FUNCTIONS | 2 | 4% |  |  |  |  |  |  |  |  |  |  |  |  |  |  |  |  |
| b730 | b730 Muscle power functions | 1 | 2% |  |  |  |  |  |  |  |  |  |  |  |  |  |  |  |  |
| b7309 | b7309 Muscle power functions, unspecified | 1 | 2% |  |  |  |  |  |  |  |  |  |  |  |  |  |  |  |  |
| b780 | b780 Sensations related to muscles and movement functions | 1 | 2% |  |  |  |  |  |  |  |  |  |  |  |  |  |  |  |  |
| b7808 | b7808 Sensations related to muscles and movement functions, other specifi | 1 | 2% |  |  |  |  |  |  |  |  |  |  |  |  |  |  |  |  |
| d | ACTIVITIES AND PARTICIPATION | 16 | 31% | 2 | 14% |  |  | 26 | 29% | 7 | 35% | 5 | 29% | 3 | 14% | 1 | 33% | 3 | 20% |
| d1 | LEARNING AND APPLYING KNOWLEDGE |  |  |  |  |  |  | 1 | 1% |  |  |  |  |  |  |  |  |  |  |
| d177 | d177 Making decisions |  |  |  |  |  |  | 1 | 1% |  |  |  |  |  |  |  |  |  |  |
| d2 | GENERAL TASKS AND DEMANDS | 6 | 12% | 2 | 14% |  |  | 5 | 5% | 5 | 25% | 1 | 6% | 2 | 9% | 1 | 33% |  |  |
| d210 | d210 Undertaking a single task | 1 | 2% |  |  |  |  |  |  |  |  |  |  |  |  |  |  |  |  |
| d2100 | d2100 undertaking a simple task | 1 | 2% |  |  |  |  |  |  |  |  |  |  |  |  |  |  |  |  |
| d230 | d230 Carrying out daily routine | 5 | 10% | 2 | 14% |  |  | 5 | 5% | 5 | 25% | 1 | 6% | 1 | 5% | 1 | 33% |  |  |
| d2301 | d2301 Managing daily routine | 4 | 8% | 2 | 14% |  |  | 3 | 3% | 3 | 15% | 1 | 6% | 1 | 5% | 1 | 33% |  |  |
| d2302 | d2302 Completing the daily routine | 1 | 2% |  |  |  |  | 2 | 2% | 1 | 5% |  |  |  |  |  |  |  |  |
| d2309 | d2309 Carrying out daily routine, unspecified |  |  |  |  |  |  |  |  | 1 | 5% |  |  |  |  |  |  |  |  |
| d299 | d299 General tasks and demands, unspecified |  |  |  |  |  |  |  |  |  |  |  |  | 1 | 5% |  |  |  |  |
| d3 | COMMUNICATION |  |  |  |  |  |  | 2 | 2% |  |  |  |  |  |  |  |  |  |  |
| d350 | d350 Conversation |  |  |  |  |  |  | 2 | 2% |  |  |  |  |  |  |  |  |  |  |
| d3501 | d3501 Sustaining a conversation |  |  |  |  |  |  | 2 | 2% |  |  |  |  |  |  |  |  |  |  |
| d4 | MOBILITY | 5 | 10% |  |  |  |  | 2 | 2% |  |  | 1 | 6% |  |  |  |  |  |  |
| d410 | d410 Changing basic body position | 2 | 4% |  |  |  |  |  |  |  |  |  |  |  |  |  |  |  |  |
| d4103 | d4103 Sitting | 1 | 2% |  |  |  |  |  |  |  |  |  |  |  |  |  |  |  |  |
| d4109 | d4109 Changing basic body position, unspecified | 1 | 2% |  |  |  |  |  |  |  |  |  |  |  |  |  |  |  |  |
| d450 | d450 Walking | 2 | 4% |  |  |  |  | 1 | 1% |  |  | 1 | 6% |  |  |  |  |  |  |
| d4500 | d4500 Walking short distances |  |  |  |  |  |  | 1 | 1% |  |  |  |  |  |  |  |  |  |  |
| d4501 | d4501 Walking long distances | 2 | 4% |  |  |  |  |  |  |  |  |  |  |  |  |  |  |  |  |
| d4509 | d4509 Walking, unspecified |  |  |  |  |  |  |  |  |  |  | 1 | 6% |  |  |  |  |  |  |
| d455 | d455 Moving around | 1 | 2% |  |  |  |  |  |  |  |  |  |  |  |  |  |  |  |  |
| d4551 | d4551 Climbing | 1 | 2% |  |  |  |  |  |  |  |  |  |  |  |  |  |  |  |  |
| d460 | d460 Moving around in different locations |  |  |  |  |  |  | 1 | 1% |  |  |  |  |  |  |  |  |  |  |
| d4609 | d4609 Moving around in different locations, unspecified |  |  |  |  |  |  | 1 | 1% |  |  |  |  |  |  |  |  |  |  |
| d5 | SELF-CARE | 3 | 6% |  |  |  |  | 1 | 1% | 1 | 5% |  |  |  |  |  |  |  |  |
| d510 | d510 Washing oneself | 1 | 2% |  |  |  |  | 1 | 1% |  |  |  |  |  |  |  |  |  |  |
| d5101 | d5101 Washing whole body | 1 | 2% |  |  |  |  | 1 | 1% |  |  |  |  |  |  |  |  |  |  |
| d540 | d540 Dressing | 1 | 2% |  |  |  |  |  |  |  |  |  |  |  |  |  |  |  |  |
| d5409 | d5409 Dressing, unspecified | 1 | 2% |  |  |  |  |  |  |  |  |  |  |  |  |  |  |  |  |
| d550 | d550 Eating | 1 | 2% |  |  |  |  |  |  | 1 | 5% |  |  |  |  |  |  |  |  |
| d6 | DOMESTIC LIFE | 1 | 2% |  |  |  |  | 5 | 5% |  |  | 2 | 12% |  |  |  |  |  |  |
| d620 | d620 Acquisition of goods and services | 1 | 2% |  |  |  |  | 1 | 1% |  |  |  |  |  |  |  |  |  |  |
| d6200 | d6200 Shopping | 1 | 2% |  |  |  |  | 1 | 1% |  |  |  |  |  |  |  |  |  |  |
| d640 | d640 Doing housework |  |  |  |  |  |  | 1 | 1% |  |  | 1 | 6% |  |  |  |  |  |  |
| d6409 | d6409 Doing housework, unspecified |  |  |  |  |  |  | 1 | 1% |  |  | 1 | 6% |  |  |  |  |  |  |
| d698 | d698 Domestic life, other specified |  |  |  |  |  |  | 2 | 2% |  |  | 1 | 6% |  |  |  |  |  |  |
| d699 | d699 Domestic life, unspecified |  |  |  |  |  |  | 1 | 1% |  |  |  |  |  |  |  |  |  |  |
| d8 | MAJOR LIFE AREAS |  |  |  |  |  |  | 2 | 2% |  |  | 1 | 6% |  |  |  |  | 2 | 13% |
| d839 | d839 Education unspecified |  |  |  |  |  |  |  |  |  |  |  |  |  |  |  |  | 1 | 7% |
| d850 | d850 Remunerative employment |  |  |  |  |  |  |  |  |  |  |  |  |  |  |  |  | 1 | 7% |
| d8509 | d8509 Remunerative employment, unspecified |  |  |  |  |  |  |  |  |  |  |  |  |  |  |  |  | 1 | 7% |
| d859 | d859 Work and employment, other specified and unspecified |  |  |  |  |  |  | 2 | 2% |  |  | 1 | 6% |  |  |  |  |  |  |
| d9 | COMMUNITY, SOCIAL AND CIVIC LIFE | 1 | 2% |  |  |  |  | 8 | 9% | 1 | 5% |  |  | 1 | 5% |  |  | 1 | 7% |
| d920 | d920 Recreation and leisure | 1 | 2% |  |  |  |  | 8 | 9% | 1 | 5% |  |  | 1 | 5% |  |  | 1 | 7% |
| d9201 | d9201 Sports |  |  |  |  |  |  | 1 | 1% |  |  |  |  |  |  |  |  |  |  |
| d9205 | d9205 Socializing |  |  |  |  |  |  | 4 | 4% | 1 | 5% |  |  |  |  |  |  |  |  |
| d9208 | d9208 Recreation and leisure, other specified |  |  |  |  |  |  | 1 | 1% |  |  |  |  |  |  |  |  |  |  |
| d9209 | d9209 Recreation and leisure, unspecified | 1 | 2% |  |  |  |  | 2 | 2% |  |  |  |  | 1 | 5% |  |  | 1 | 7% |
| e | ENVIRONMENTAL FACTORS |  |  | 1 | 7% |  |  |  |  | 1 | 5% |  |  |  |  |  |  |  |  |
| e3 | SUPPORT AND RELATIONSHIPS |  |  |  |  |  |  |  |  | 1 | 5% |  |  |  |  |  |  |  |  |
| e399 | e399 Support and relationships, unspecified |  |  |  |  |  |  |  |  | 1 | 5% |  |  |  |  |  |  |  |  |
| e4 | ATTITUDES |  |  | 1 | 7% |  |  |  |  |  |  |  |  |  |  |  |  |  |  |
| e499 | e499 Attitudes, unspecified [close people] |  |  | 1 | 7% |  |  |  |  |  |  |  |  |  |  |  |  |  |  |
| nc/nd |  | 2 | 4% |  |  |  |  |  |  |  |  |  |  | 8 | 36% |  |  |  |  |

| **Frequency of meaningful concepts per scale** |
| --- |
| **Figure S1: ICF linking per scale for multi-scale instruments** |

| **Table S2: ICF Ratings per scale for the multi-scale instruments** | | | | | | | | | | | | | | | | | |
| --- | --- | --- | --- | --- | --- | --- | --- | --- | --- | --- | --- | --- | --- | --- | --- | --- | --- |
|  | **b1300 Energy level** | **b1301 Motivation** | **b1400 Sustaining attention** | **b1409 Attention functions,** | **b1528 Emotional functions, other specified** | **b1608 Thought functions, other specified** | **b1609 Thought functions, unspecified** | **b4552 Fatiguability** | **d2301 Managing daily routine** | **d2309 Carrying out daily routine, unspecified** | **d299 General tasks and demands, unspecified** | **d839 Education unspecified** | **d8509 Remunerative employment, unspecified** | **d9209 Recreation and leisure, unspecified** | **e499 Attitudes, unspecified [close people]** | **nd-gh** | **nd-ph** |
| PFS - Affective | 3 |  |  |  |  |  |  |  |  |  |  |  |  |  |  |  |  |
| PFS - Behavioural | 3 |  |  |  |  |  |  |  |  |  |  | 1 | 1 | 1 |  |  |  |
| PFS – Cognitive / Mood |  |  |  | 1 | 1 |  | 1 |  |  |  |  |  |  |  |  |  |  |
| PFS - Sensory | 3 |  |  |  |  |  |  |  |  |  |  |  |  |  |  |  |  |
| MFI - General fatigue | 2 |  |  |  |  |  |  | 1 |  |  |  |  |  |  |  | 1 |  |
| MFI - Mental fatigue |  |  | 4 |  |  |  |  |  |  |  |  |  |  |  |  |  |  |
| MFI - Physical fatigue |  |  |  |  |  |  |  | 2 |  |  |  |  |  |  |  |  | 2 |
| MFI - Reduced activity |  |  |  |  |  |  |  |  |  | 4 |  |  |  |  |  |  |  |
| MFI - Reduced motivation |  | 1 |  |  | 1 |  |  |  | 1 |  | 1 |  |  | 1 |  |  |  |
| PRO-CTCAE - Interference | 1 |  |  |  |  |  |  |  | 1 |  |  |  |  |  |  |  |  |
| PRO-CTCAE - Severity | 1 |  |  |  |  |  |  |  |  |  |  |  |  |  |  |  |  |
| QLQ-FA12 - Social sequalae | 1 |  |  |  |  |  |  |  |  |  |  |  |  |  | 1 |  |  |
| QLQ-FA12 - Interference with daily life | 1 |  |  |  |  |  |  |  | 1 |  |  |  |  |  |  |  |  |
| QLQ-FA12 - Emotional Fatigue |  |  |  |  | 3 |  |  |  |  |  |  |  |  |  |  |  |  |
| QLQ-FA12 - Cognitive Fatigue |  |  |  |  |  | 2 |  |  |  |  |  |  |  |  |  |  |  |
| QLQ-FA12 - Physical Fatigue | 4 |  |  |  |  |  |  |  | 1 |  |  |  |  |  |  |  |  |

|  |
| --- |
| **Figure S2: Types of fatigue per scale for multi-scale instruments** |

| **Table S3: Types of fatigue per scale for multi-scale instruments** | | | | | |
| --- | --- | --- | --- | --- | --- |
|  | cognitive | emotional | general | other | physical |
| PFS - Affective |  |  | 3 |  |  |
| PFS - Behavioural |  |  | 3 |  |  |
| PFS - Cognitive/Mood | 2 | 1 |  |  |  |
| PFS - Sensory |  |  | 1 |  | 2 |
| MFI - General fatigue |  |  | 3 |  | 1 |
| MFI - Mental fatigue | 4 |  |  |  |  |
| MFI - Physical fatigue |  |  |  |  | 4 |
| MFI - Reduced activity |  |  | 4 |  |  |
| MFI - Reduced motivation |  | 5 |  |  |  |
| PRO-CTCAE - Interference |  |  | 1 |  |  |
| PRO-CTCAE - Severity |  |  | 1 |  |  |
| QLQ-FA12 - Social sequalae |  |  |  | 1 |  |
| QLQ-FA12 – Interference with daily life |  |  | 1 |  |  |
| QLQ-FA12 - Emotional Fatigue |  | 3 |  |  |  |
| QLQ-FA12 - Cognitive Fatigue | 2 |  |  |  |  |
| QLQ-FA12 - Physical Fatigue |  |  | 3 |  | 2 |
